# Myricetin Ameliorates Defective Post-Receptor Insulin Signaling via *β*-Endorphin Signaling in the Skeletal Muscles of Fructose-Fed Rats

**DOI:** 10.1093/ecam/neq017

**Published:** 2011-06-04

**Authors:** Thing-Fong Tzeng, Shorong-Shii Liou, I-Min Liu

**Affiliations:** ^1^Department of Internal Medicine, Pao Chien Hospital, Ping Tung City, China; ^2^Department of Pharmacy & Graduate Institute of Pharmaceutical Technology, Tajen University, Yen-Pou, Ping Tung Shien, Taiwan

## Abstract

**β**-Endorphin plays a major role in the amelioration of insulin resistance. The present study documents that myricetin (3,5,7,3′, 4′, 5′-hexahydroxyflavone) ameliorates insulin resistance by enhancing **β**-endorphin production in insulin-resistant rats. The rats were induced for insulin resistance by feeding them a diet containing 60% fructose for 6 weeks. The degree of insulin resistance was measured by the homeostasis model assessment of basal insulin resistance (HOMA-IR). The plasma levels of insulin and **β**-endorphin were measured by an enzyme-linked immunosorbent assay. The insulin receptor-related signaling mediators in the soleus muscles of rats were evaluated by immunoprecipitation or immunoblotting. Myricetin was injected daily (1 mg kg^−1^ per injection, thrice daily) for 14 days. Consequently, the high-glucose plasma levels in fructose-fed rats decreased significantly concomitant with an increase in plasma **β**-endorphin. The reduction of the elevated HOMA-IR index following treatment with myricetin was subsequently inhibited by the administration of **β**-funaltrexamine hydrochloride (**β**-FNA) at doses sufficient to block **μ**-opioid receptors (MOR). The myricetin treatment was also observed to affect the phosphorylation of the insulin receptor, insulin receptor substrate-1, Akt and Akt substrate of 160 kDa, with subsequent effects on glucose-transporter subtype 4 translocation, all of which were blocked by **β**-FNA pretreatment. These results indicated that enhancement of **β**-endorphin secretion, which in turn leads to peripheral MOR activation, is involved in the action of myricetin on the amelioration of impaired signaling intermediates downstream of insulin receptors.

## 1. Introduction

Myricetin (3,5,7,3′, 4′, 5′-hexahydroxyflavone) is a flavonoid commonly found in tea, berries, fruits, vegetables and medicinal herbs. Myricetin has been shown to possess antioxidative and cytoprotective properties [1, 2]. A therapeutic effect of myricetin in patients with cardiovascular diseases associated with diabetes mellitus has also been reported [1, 2]. An insulinomimetic effect of myricetin on lipogenesis and glucose transport in the adipocytes of rats with noninsulin-dependent diabetes mellitus has been observed [3]. Myricetin has been found to reduce hyperglycemia in diabetic rats, possibly through its ability to increase hepatic glycogen synthesis and to normalize hypertriglyceridemia [4]. Myricetin has also demonstrated the ability to improve glucose utilization, lowering plasma-glucose levels in a type 1 diabetes-like animal model [5]. Furthermore, myricetin displays the characteristics of rosiglitazone, demonstrating improved glucose utilization and ameliorating the impaired insulin-signaling pathway in insulin-resistant rats induced by the high intake of fructose. However, unlike rosiglitazone, it does not cause an increase in body weight [6], suggesting that myricetin can serve as a therapeutic adjunct for the treatment of insulin-resistant patients and/or for patients susceptible to the thiazolidinediones-induced side effects of weight gain and edema [7].

Insulin resistance in genetically obese Zucker rats was shown to improve upon exercise, and *β*-endorphin is implicated in this exercise-induced improvement of insulin resistance [8]. The *β*-endorphin has been shown to reverse the impaired insulin-stimulated glucose disposal in insulin-resistant rats induced by a fructose-rich diet [9]. Furthermore, it was documented that activation of *μ*-opioid receptors (MOR) by loperamide reversed insulin-stimulated glucose uptake impaired by cytokines in C_2_C_12_ cells, with a marked recovery of insulin signaling [10], and can ameliorate defective postreceptor insulin signaling, reversing the impairment of insulin-stimulated glucose utilization in obese Zucker rats [11]. A direct role has been demonstrated for peripheral MOR activation in the improvement of insulin resistance induced by a high intake of fructose, as this improvement is more rapid in MOR knockout mice than in wild-type mice [12]. These results led to the idea that activation of MOR on insulin-targeted organs may have a beneficial effect in reducing insulin resistance.

We have shown that activation of MORs on the soleus muscle, in response to increased endogenous *β*-endorphin secretion, is essential to the plasma glucose-lowering action of myricetin in diabetic rats lacking insulin [13]. This study was undertaken to ascertain whether elevation of circulating *β*-endorphin and/or activation of peripheral MORs could mediate the action of myricetin and ameliorate insulin resistance.

## 2. Methods

### 2.1. Materials

Standard rat chow containing 60% vegetable starch, 5% fat and 18% protein (Cat. #2018) and fructose-rich rat chow containing 60% fructose, 5% fat and 18% protein (Cat. #TD 89247) were obtained from Harlan Teklad (Madison, WI, USA). Myricetin (purity >96%; Cat. #M6760) and protein A-sepharose beads were purchased from Sigma-Aldrich, Inc. (St. Louis, Missouri, USA). *β*-funaltrexamine hydrochloride (*β*-FNA; Cat. #0926) was purchased from Tocris Bioscience (Bristol, UK). The diagnostic kit used for determination of plasma glucose levels (Cat. #COD12503) was purchased from BioSystem (Costa Brava, Barcelona, Spain). The rat insulin enzyme-linked immunosorbent assay (ELISA) kit was obtained from LINCO Research, Inc. (St. Charles, MO, USA; Cat. #EZRMI-13K). The ELSA kit used for assaying *β*-endorphin-like immunoreactivity (BER) was obtained from Peninsula Laboratories Inc. (Belmont, CA, USA; Cat. #T-4040). The kit used for the protein dye-binding assay was purchased from Bio-Rad Laboratories (CA, USA). Anti-insulin receptor (IR) *β*-subunit (Cat. #MS-634 for immunoprecipitation; Cat. #MS-636 for western blotting), anti-insulin receptor substrate (IRS)-1 (Cat. #MS-630) and anti-phosphotyrosine (Cat. #MS-445) antibodies were obtained from NeoMarkers (Fremont, CA, USA). Anti-p85 subunit of phosphatidylinositol (PI) 3-kinase (p85) (Cat. #4292), anti-Akt (Cat. #9272), anti-phospho-Ser^473^Akt (Cat. #9271), anti-phospho-Thr^308^Akt, anti-phospho-(Ser/Thr) Akt substrate (Cat. #9611) and anti-glucose-transporter subtype 4 (GLUT 4) (Cat. #2299) antibodies were acquired from Cell Signaling Technology, Inc. (Beverly, MA, USA). The anti-AS160 (Rab GAP) antibody was purchased from Upstate (Charlottesville, VA; Cat. #07-741). ECL Western Blotting Systems were obtained from Amersham Corp. (Braunschweig, Germany). Bovine insulin was obtained from Novo Nordisk (Bagsvaerd, Denmark). All other reagents were obtained from standard sources.

### 2.2. Animal Models

Eight-week-old, male Wistar rats were obtained from the National Laboratory Animal Center (Taipei, Taiwan). They were maintained in a temperature-controlled room (25 ± 1°C) and kept on a 12 : 12 light-dark cycle (light on at 06:00 h) in our animal center. Rats received the fructose-rich rat chow for six additional weeks to induce insulin resistance [6]. Food and water were available *ad libitum*. All animal procedures were performed according to the Guide for the Care and Use of Laboratory Animals of the National Institutes of Health as well as the guidelines of the Animal Welfare Act.

### 2.3. Treatment Procedures

The fructose-fed rats were divided into three experimental groups. It has been documented that fructose-fed rats receiving intravenous (i.v.) injections of myricetin in the lateral tail vein every 8 h, thrice daily (at 06:00., 14:00 and 22:00 h) at 1 mg kg^−1^ per injection for 14 consecutive days were found to demonstrate effectively ameliorated insulin resistance [6]. Thus, one group of fructose-fed rats was given i.v. injections of 1 mg kg^−1^ myricetin according to the treatment regimen described above. The second group of fructose-fed rats received injection of myricetin plus *β*-FNA [14]. *β*-FNA was i.v. injected 30 min prior to administration of myricetin. The remaining fructose-fed rats given the same volume of vehicle (saline) used to dissolve the test medications were considered as the vehicle-treated group. The injection volume was controlled in that 1 mL kg^−1^ was routinely administered. The injection was given slowly to avoid as much pain and shock as possible and thereby helping to exclude non-predictive outcomes. The rats were maintained on a fructose diet during the treatment period. Water was made available *ad libitum* throughout the experiment.

After a 2-week treatment, blood samples (0.1 mL) were collected from the tail vein of rats when deeply anesthetized by sodium pentobarbital (30 mg kg^−1^, intraperitoneal). Blood samples were used to evaluate glucose, insulin and *β*-endorphin plasma levels. The homeostasis model assessment of basal insulin resistance (HOMA-IR) was used to calculate an index from the product of the fasting concentrations of plasma glucose (mmol L^−1^) and plasma insulin (*μ*U mL^−1^) divided by 22.5 [15]. Lower HOMA-IR values indicated greater insulin sensitivity, whereas higher HOMA-IR values indicated lower insulin sensitivity (insulin resistance).

### 2.4. Plasma Analysis

Plasma glucose concentrations were measured using the glucose oxidase method by means of a commercially available kit. The ELISA technique was employed to quantify the plasma levels of insulin and BER. All samples were analyzed in triplicate.

### 2.5. In Vivo Insulin Receptor Activation

To assess the changes in insulin-receptor activation *in vivo*, rats in the fed state were anesthetized with sodium pentobarbital at the end of the 2-week treatment period. A bolus of insulin (10 U kg^−1^) was then injected into the portal vein, as described previously [6]. Approximately 120 s after insulin injection, rats were sacrificed and the soleus muscle was immediately extirpated, washed with cold phosphate buffer and cut into 200–300 mg portions, which were then stored separately at −80°C for subsequent experiments.

### 2.6. Muscle Processing

Cytosol and membrane fractions were prepared according to the previous method [6, 16]. Briefly, muscles used for measuring insulin signaling were weighed while still frozen and homogenized (Polytron, Brinkmann Instruments, Inc., Westbury, NY, USA) in 0.4 mL homogenizing buffer containing 250 mmol L^−1^ sucrose, 20 mmol L^−1^ Tris (pH: 7.5), 2 mmol L^−1^ ethylenediaminetetraacetic acid (EDTA), 0.5 mmol L^−1^ ethylene glycol tetraacetic acid (EGTA), 20 *μ*g mL^−1^ leupeptin, 10 *μ*g mL^−1^ aprotinin, 174.2 *μ*g L^−1^ phenylmethylsulfonyl fluoride and 20 mmol L^−1^ dithiothreitol. The homogenate was centrifuged at 100 000 g for 1 h at 4°C. The supernatant (cytosolic extract) was transferred to a tube kept on ice, whereas the pellet was resuspended in 0.45 mL homogenizing buffer containing 5% Triton X-100. The resuspended pellet fraction was then centrifuged at 14 000 g  for 5 min at 4°C, and the pellet was discarded. The supernatant from this spin constitutes the membrane extract. Protein concentrations were determined by the BioRad protein dye-binding assay. The supernatant was stored at −80°C until used in immunoprecipitation and Western immunoblotting.

### 2.7. Immunoprecipitation

Muscle lysates (500 *μ*g) were subjected to immunoprecipitation with the anti-IR *β*-subunit, anti-IRS-1 or anti-AS160 (Rab GAP) antibody at 4°C overnight, followed by shaking with protein A-Sepharose beads for 1 h. The bead-Protein A-antibody-antigen complexes were precipitated by brief centrifugation. The pellets were washed thrice in ice-cold buffer (0.5% Triton X-100, 100 mmol L^−1^ Tris, pH: 7.4, 10 mmol L^−1^ EDTA and 2 mmol L^−1^ sodium vanadate), resuspended in Laemmli sample buffer and boiled for 5 min. The sepharose beads were precipitated by brief centrifugation and the supernatant prepared for sodium dodecyl sulfate-polyacrylamide gel electrophoresis (SDS-PAGE, 10% acrylamide gel) using a Bio-Rad Mini-Protein II system (55 and 130 V during the stacking and separation phases, respectively). Protein was transferred to a polyvinylidene difluoride (PVDF) membrane using a Bio-Rad Trans-Blot system (2 h at 20 V in 25 mmol L^−1^ Tris, 192 mmol L^−1^ glyceine and 20% methanol (MeOH)). Following transfer, the membrane was probed with anti-IR *β*-subunit, anti-IRS-1 and anti-phosphotyrosine antibodies.

### 2.8. Immunoblotting

Equal amounts of protein (50 *μ*g) were prepared from muscle homogenates, subjected to SDS-PAGE, transferred to PVDF membrane as described above and probed with anti-PI3-kinase p85, anti-Akt and anti-GLUT 4 antibodies according to the manufacturer's instructions. Protein phosphorylation of Akt was measured using the anti-phospho-Ser^473^Akt and anti-phospho-Thr^308^Akt antibodies. Phosphorylation of Akt substrate of 160 kDa (PAS-AS160) was detected using the anti-phospho-(Ser/Thr) Akt substrate antibody. PAS-AS160 recognizes Akt phosphorylation-motif peptide sequences (RXRXXpT/S). After three 5-min washes in Tris Buffered Saline Tween-20 (TBST; 20 mmol L^−1^ Tris-HCl, pH: 7.5, 150 mmol L^−1^ sodium chloride (NaCl) and 0.05% Tween 20), membranes were incubated with the appropriate peroxidase-conjugated secondary antibodies. The membranes were then washed thrice in TBST and visualized on X-ray film using the ECL Western Blotting System. Densities of the obtained immunoblots were quantified using a laser densitometer. The mean value for samples from the vehicle-treated group on each immunoblot, expressed in densitometry units, was adjusted to a value of 1. All experimental sample values were then expressed relative to this adjusted mean value.

### 2.9. Statistical Analysis

Data are expressed as the mean ± SEM for each group of animals at the number indicated in the tables. Statistical differences among groups were determined using two-way repeated-measures analysis of variance (ANOVA). Dunnett range *post hoc* comparisons were used to determine the source of significant differences where appropriate. A *P*-value < .05 was considered statistically significant.

## 3. Results

### 3.1. General Characteristics of Fructose-Fed Rats

Following the injection of myricetin, plasma glucose levels in fructose-fed rats fell to a value significantly lower than in the vehicle-treated group (*P* < .05), showing a plasma glucose-lowering activity of 18.1 ± 3.2% (Table 1). Meanwhile, an elevation of the plasma BER level in fructose-fed rats was observed following myricetin treatment (Table 1). In the presence of *β*-FNA (10 *μ*g kg^−1^), the plasma glucose-lowering action of myricetin in fructose-fed rats was eliminated to approach the values of the vehicle-treated group (Table 1). Myricetin-induced secretion of plasma BER was unaffected in fructose-fed rats pretreated with *β*-FNA at any dosage (Table 1). Additionally, the HOMA-IR score in fructose-fed rats receiving 2 weeks of myricetin treatment showed a decrease of *∼*70% of the score observed in the vehicle-treated group (Table 1). Pretreated fructose-fed rat with *β*-FNA (10 *μ*g kg^−1^) was observed to abolish this myricetin-induced reduction of the HOMA-IR (Table 1).

### 3.2. Protein Levels and Degrees of Insulin Receptor (IR) and Insulin Receptor Substrate (IRS)-1 Tyrosine Phosphorylation

**Figure 1:**
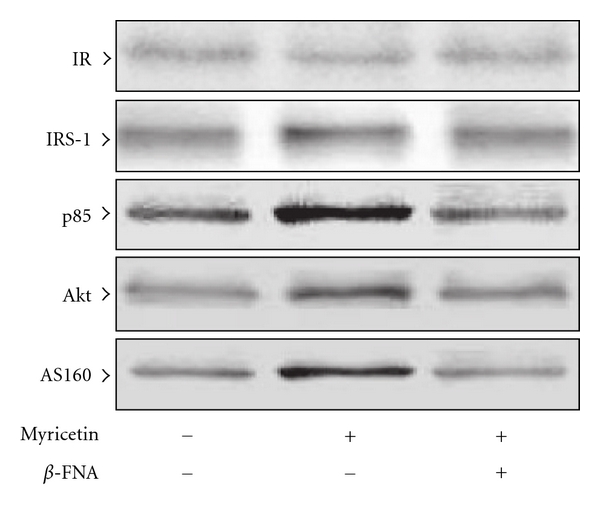
Protein expression of insulin receptor-related signaling mediators in the soleus muscles of fructose-fed rats receiving 14-day treatment with myricetin (1 mg kg^−1^ per i.v. injection, thrice daily) or myricetin plus *β*-FNA. *β*-FNA (10 *μ*g kg^−1^ per injection) was i.v. injected 30 min prior to administration of myricetin. Rats that did not receive any treatment were given the same volume of vehicle used to dissolve the test medications. Findings were reproduced on four separate occasions. The quantification of the data is shown in Table 2.

There were no significant differences in the expression of IR protein in the soleus muscle of fructose-fed rats between any of the groups (Figure 1). Two-week myricetin treatment slightly elevated the levels of IRS-1 protein expression in the soleus muscles of fructose-fed rats, and this was antagonized by *β*-FNA (Figure 1). The quantification of the immunoblots is summarized in Table 2.

**Figure 2:**
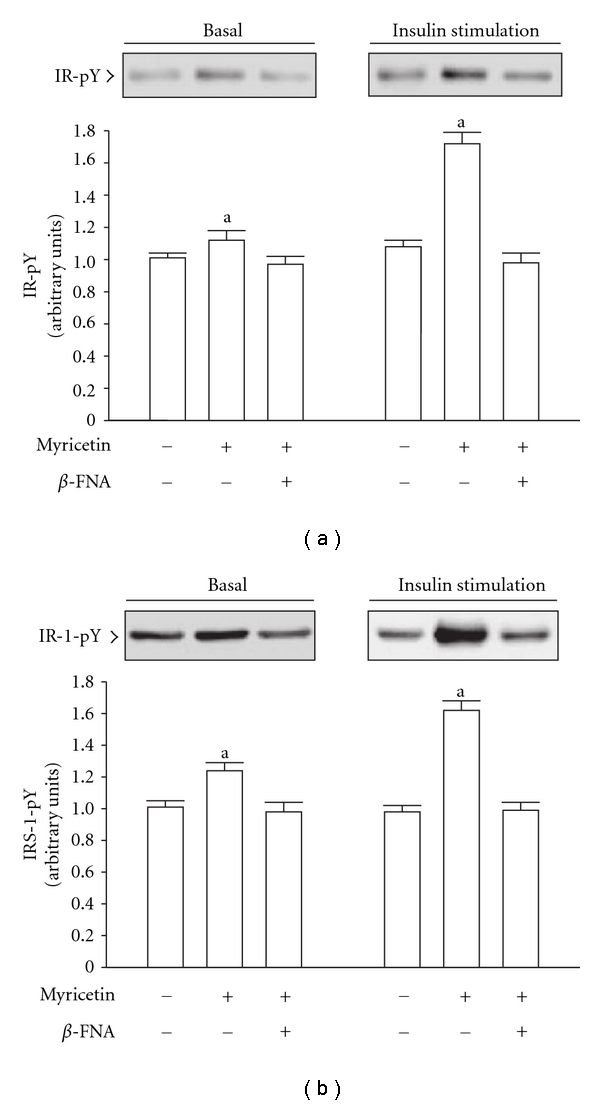
Tyrosine phosphorylation (pY) of insulin receptor (IR)(a) and insulin receptor substrate (IRS)-1(b) in the soleus muscles of fructose-fed rats receiving 14-day treatment with myricetin (1 mg kg^−1^ per i.v. injection, thrice daily) or myricetin plus *β*-FNA. *β*-FNA (10 *μ*g kg^−1^ per injection) was i.v. injected 30 min prior to administration of myricetin. Rats that did not receive any treatment were given the same volume of vehicle used to dissolve the test medications. Findings were reproduced on four separate occasions. Quantification of protein levels are expressed as mean ± SEM (*n* = 5 per group) in each column. ^a^ *P* < .05 represents the level of significance compared to the basal values of the vehicle-treated group.

Under non-insulin stimulating conditions, the degree of tyrosine phosphorylation of IR and IRS-1 in the soleus muscles of fructose-fed rats was slightly elevated following the 2-week myricetin treatment, but these myricetin-induced effects were not observed in rats pretreated with *β*-FNA (Figure 2). The extent of IR and IRS-1 tyrosine phosphorylation in the soleus muscles of fructose-fed rats in response to insulin stimulation was markedly elevated in the 2-week myricetin-treated group. Myricetin failed to induce similar changes in IR and IRS-1 tyrosine phosphorylation in the soleus muscles of fructose-fed rats pretreated with *β*-FNA (Figure 2).

### 3.3. Changes in Protein Levels and Amount of the p85 Subunit of PI3-Kinase Associated with IRS-1

Two-week treatment of fructose-fed rats with myricetin improved the expression of the p85 subunit of PI3-kinase in the soleus muscles, and this effect was abolished by *β*-FNA pretreatment (Figure 2). The quantification of the immunoblots is summarized in Table 2.

**Figure 3:**
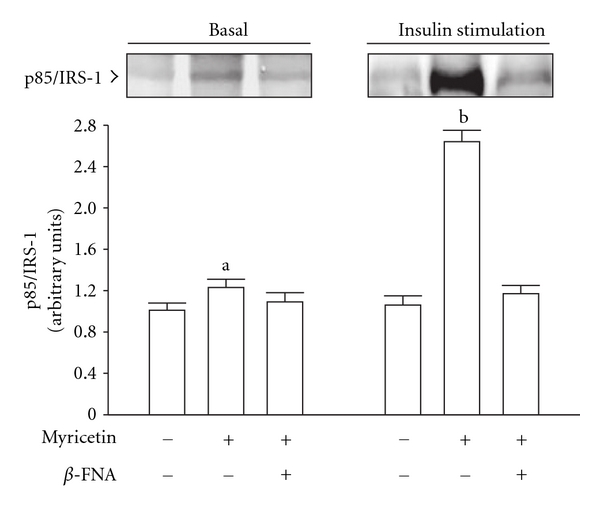
The amount of the p85 subunit of PI3-kinase associated with IRS-1(p85/IRS-1) in the soleus muscles of fructose-fed rats receiving 14-day treatment with myricetin (1 mg kg^−1^ per i.v. injection, thrice daily) or myricetin plus *β*-FNA. *β*-FNA (10 *μ*g kg^−1^ per injection) was i.v. injected 30 min prior to administration of myricetin. Rats that did not receive any treatment were given the same volume of vehicle used to dissolve the test medications. Findings were reproduced on four separate occasions. Quantification of protein levels expressed as mean ± SEM (*n* = 5 per group) in each column. ^a^ *P* < .05 and ^b^ *P* < .01 represent the level of significance compared to the basal values of the vehicle-treated group.

Following a 2-week myricetin treatment, the basal degree of p85 associated with IRS-1 in the soleus muscles of fructose-fed rats was slightly higher than the levels of the vehicle-treated controls, and myricetin failed to induce similar changes in fructose-fed rats pretreated with *β*-FNA (Figure 3). The higher degree of insulin-stimulated p85 associated with IRS-1 in myricetin-treated rats was clearly prevented by *β*-FNA pretreatment (Figure 3).

### 3.4. Protein Levels and Degree of Akt and Akt Substrate of 160 kDa (AS160) Phosphorylation

After 2 weeks of myricetin treatment, the basal protein levels of Akt and AS160 in the soleus muscles of fructose-fed rats were higher to those of their vehicle-treated group, and this difference was eliminated by *β*-FNA pretreatment (Figure 2). The quantification of the immunoblots is summarized in Table 2.

**Figure 4:**
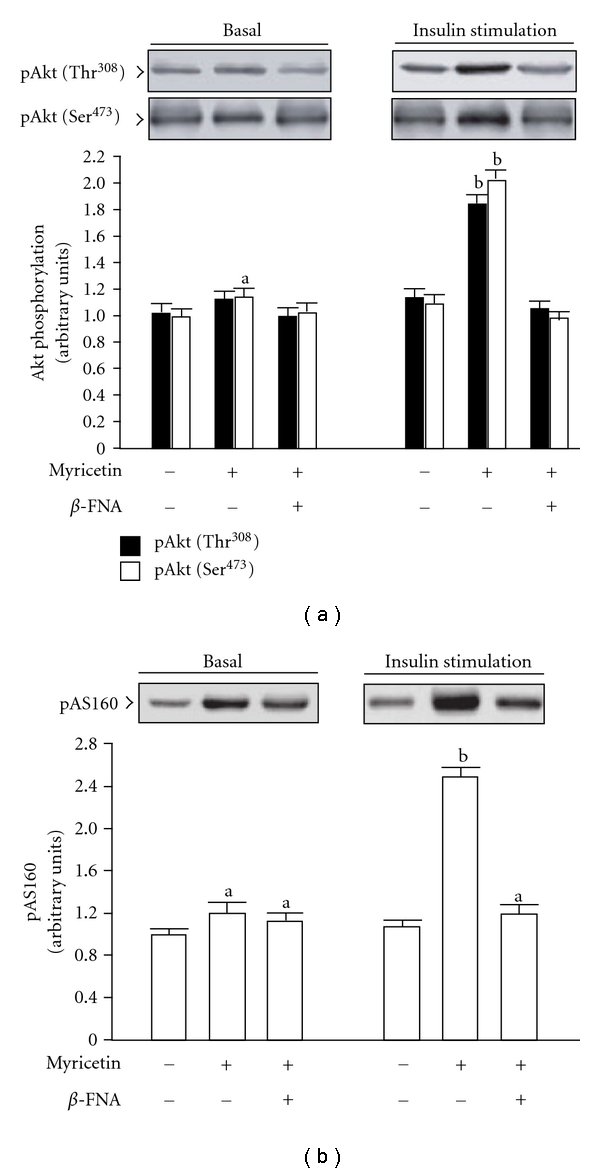
Phosphorylation of Akt (a) and AS160 (b) in the soleus muscles of fructose-fed rats receiving 14-day treatment with myricetin (1 mg kg^−1^ per i.v. injection, thrice daily) or myricetin plus *β*-FNA. *β*-FNA (10 *μ*g kg^−1^ per injection) was i.v. injected 30 min prior to administration of myricetin. Rats that did not receive any treatment were given the same volume of vehicle used to dissolve the test medications. Findings were reproduced on four separate occasions. Quantification of protein levels expressed as mean ± SEM (*n* = 5 per group) in each column. ^a^ *P* < .05 and ^b^ *P* < .01 represent the level of significance compared to the basal values of the vehicle-treated group.

It was observed that the degree of basal phosphorylation of Akt (Thr^308^/Ser^473^) and AS160 was increased in the soleus muscles of fructose-fed rats receiving 2-week myricetin treatment (Figure 4). The degree of insulin-stimulated phosphorylation of Akt tyrosine (Thr^308^) and Akt serine (Ser^473^) was also significantly elevated by myricetin to *∼*1.8-fold and 2.2-fold relative to that of the vehicle-treated controls, respectively (Figure 4(a)). Furthermore, the degree of insulin-stimulated AS160 phosphorylation in the soleus muscles of myricetin-treated fructose-fed rats was markedly higher than the value of their vehicle-treated group (Figure 4(b)). These myricetin-induced effects were eliminated by *β*-FNA pretreatment (Figure 4).

### 3.5. Insulin-Stimulated Glucose-Transporter Subtype 4 (GLUT 4) Translocation

**Figure 5:**
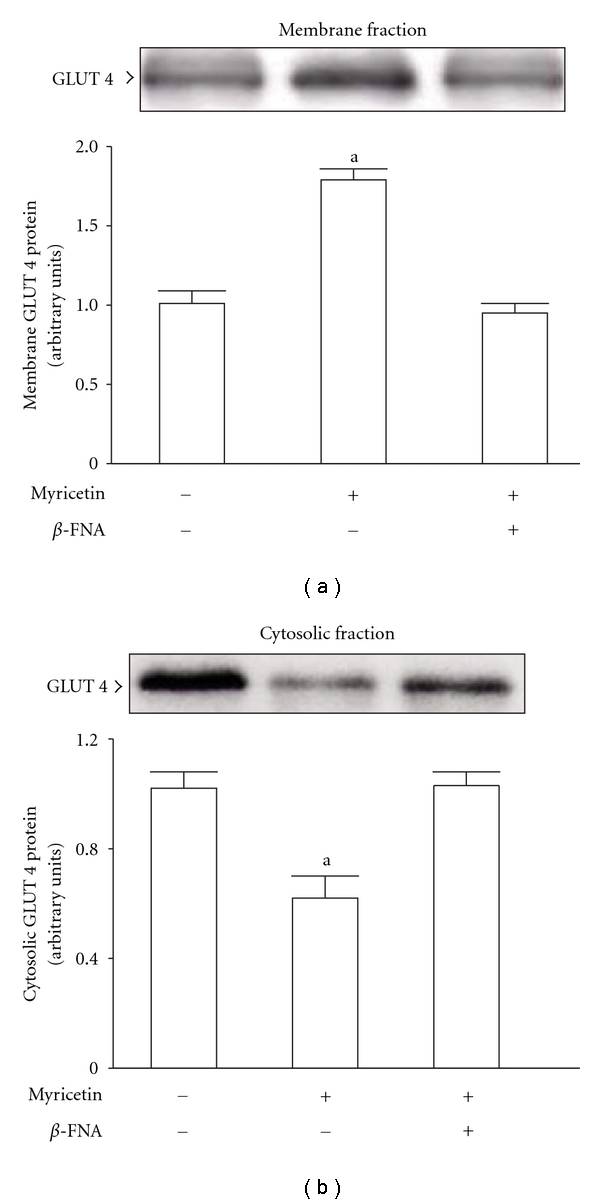
Insulin-stimulated GLUT 4 protein expression in the membrane (a) and cytosolic (b) fractions in the soleus muscles of fructose-fed rats receiving 14-day treatment with myricetin (1 mg kg^−1^ per i.v. injection, thrice daily) or myricetin plus *β*-FNA. *β*-FNA (10 *μ*g kg^−1^ per injection) was i.v. injected 30 min prior to administration of myricetin. Rats that did not receive any treatment were given the same volume of vehicle used to dissolve the test medications. Findings were reproduced on four separate occasions. Quantification of protein levels expressed as mean ± SEM (*n* = 5 per group) in each column. ^a^ *P* < .05 represents the level of significance compared to the values of the vehicle-treated group.

At the termination of the 2-week myricetin treatment, insulin-stimulated GLUT 4 protein expression in the membrane fraction of the soleus muscles from fructose-fed rats was increased *∼*1.8-fold of that observed in their vehicle-treated group; conversely, the protein levels in the cytosolic fraction of the same samples decreased to 60% of that observed in the vehicle-treated controls (Figure 5). The myricetin-mediated changes in insulin-stimulated GLUT 4 translocation were reversed by *β*-FNA pretreatment (Figure 5).

## 4. Discussion

In the present study, a 2-week treatment regimen with myricetin (1 mg kg^−1^ per i.v. injection, thrice daily) was found to significantly increase plasma BER in a manner that paralleled the lowering of plasma glucose in rats fed for 6 weeks with fructose chow, highlighting the possibility that increased plasma BER and the reduction of plasma glucose concentrations are related phenomena in this model. Although the effect of myricetin on the lowering of plasma glucose was suppressed by blockade of MOR via *β*-FNA pretreatment, this antagonist had no influence on the myricetin-induced secretion of plasma BER. As illustrated by the findings that myricetin promoted an increase in plasma BER concurrent with a lowering of plasma glucose concentrations, a direct effect of this flavonol on glucose homeostasis in insulin-resistant rats seems unlikely.

In humans and other mammals, skeletal muscle normally accounts for *∼*75% of whole-body, insulin-stimulated glucose transport [17]. An impaired ability of skeletal muscles to respond to insulin is, therefore, disruptive to systemic glucose homeostasis. Actually, the increase in insulin action on skeletal muscle is likely to be related to increased protein expression and/or functional activity of several key components of the insulin signal-transduction pathway; defects in the insulin-signaling cascade, which lead to impaired glucose utilization, are believed to play a key role in the pathogenesis of insulin resistance [18]. It is conceivable that IRS-1 tyrosine phosphorylation (in response to insulin stimulation) generally increases the association of IRS-1 with the p85 subunit of PI3-kinase, resulting in increased PI3-kinase activity. In turn, this would lead to the activation of serine/threonine kinase Akt (protein kinase B) and, ultimately, accelerated rates of GLUT 4 translocation, such that GLUT 4 manifests predominantly at the cell surface and enhances insulin-stimulated glucose disposal [19]. It has been documented that the action of insulin on glucose uptake and metabolism is much greater in skeletal muscles composed primarily of oxidative fibers (e.g., the soleus) as compared to glycolytic fibers (e.g., the epitrochlearis and extensor digitorum longus), even though the soleus muscle represents a small portion of the total muscle mass [20]. Activation of MOR located on the soleus muscle, resulting in reversal of the impairment of insulin-stimulated glucose disposal in obese Zucker rats, has been documented; this improvement in insulin resistance was associated with the amelioration of the post-receptor insulin-signaling cascade, including downstream effectors of the PI3-kinase signaling pathway involved in glucose-transporter translocation [11]. We found that increasing the doses of *β*-FNA negatively correlated with the effect of myricetin on the reduction of higher HOMA-IR in fructose-fed rats, although the higher plasma levels of BER were still apparent in the myricetin-treated group. This raised the possibility that an elevation of circulating *β*-endorphin and/or activation of peripheral MOR might be the mechanisms by which myricetin ameliorates the defective insulin action. With this in mind, soleus muscle samples were prepared from all animals after insulin stimulation.

A substrate of Akt has been identified that may provide a link between insulin signaling and GLUT 4 trafficking. This protein, AS160, has a GAP homology domain and is phosphorylated by Akt in response to insulin [21]. In this study we showed that myricetin treatment had clear effects on the IRS-1/PI3-kinase/Akt/AS160 signaling cascade (at both the protein expression and phosphorylation levels) and subsequent GLUT 4 trafficking, which were all suppressed by MOR antagonism. These findings suggested that the effect of myricetin on the improvement of post-receptor insulin signaling might be dependent on the activation of MOR located on peripheral insulin-sensitive tissues, such as skeletal muscle. Additionally, these results supported the essential role of opioids during glucose homeostasis. Furthermore, opioids or opioid receptor activation, especially of the *μ*-subtype, may have key functions in glycemic control.

**Figure 6:**
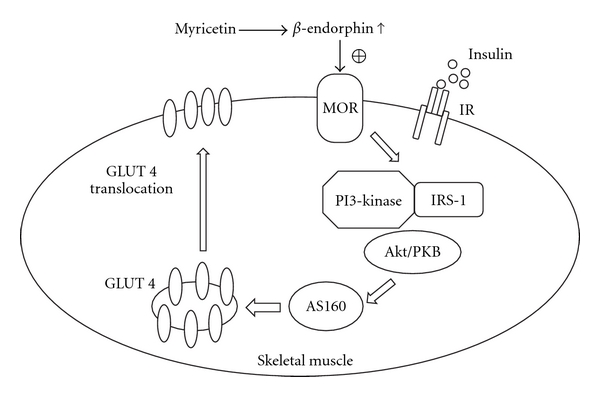
The possible mechanisms of myricetin action on the amelioration of defective post-receptor insulin signaling in the skeletal muscle of fructose-fed rats.

Although *β*-endorphin is released with adrenocorticotropic hormone from the pituitary gland [22], the pituitary gland-independent release of endogenous opioids occurs in other organs, such as the adrenal gland [9, 23]. We provided new evidence that activation of *α*_1_-adrenoceptors on the adrenal gland may increase *β*-endorphin secretion via the phospholipase C-protein kinase C pathway, which in turn activates peripheral MOR to modify glucose metabolism-associated genes, thereby leading to improved peripheral glucose utilization and decreased hepatic gluconeogenesis for amelioration of severe hyperglycemia in type 1-like diabetes [24]. Secretion of *β*-endorphin from the adrenal gland was observed to be essential to the plasma glucose-lowering action of myricetin in streptozotocin-induced (STZ)-diabetic rats [13]. It is not clear if myricetin-induced *β*-endorphin secretion in fructose-fed rats is mediated by pituitary gland-dependent or -independent pathways; however, it was strongly demonstrated that the myricetin-mediated action converged at a point between the downstream targets of the peripheral MOR and the IRS-PI3-kinase signaling pathway, subsequently affecting insulin-induced GLUT 4 translocation (Figure 6).

A widely recommended approach for the control of hyperglycemia is to administer a parenteral insulin preparation that provides a constant source of circulating insulin for 12–24 h to augment or replace deficient endogenous insulin secretion, although insulin resistance is eventually developed in these insulin-treated patients [25]. The mechanisms underlying the concentration- and time-dependent induction of insulin resistance are complex and incompletely defined. Thus, it is imperative to identify new targets that can achieve equal and/or superior effects on the control of glycemia with a reduction in insulin resistance. The findings of our study provide new insights into the chemical compounds or herbal products that might enhance *β*-endorphin secretion and/or stimulate MOR in peripheral insulin-sensitive tissue, which might serve as potential agents or adjuvants for targeting glucose metabolism in insulin-resistant individuals.

## 5. Conclusion

The survey showed that the action of myricetin on the amelioration of defective post-receptor insulin signaling, especially as it related to IRS-1-associated PI3-kinase and GLUT 4 translocation, required *β*-endorphin as a mediator to activate peripheral MOR.

## Funding

National Science Council Grant (NSC 94-2320-B-127-005) of Taiwan, Republic of China.

## Figures and Tables

**Table 1:** General characteristics of fructose-fed rats after 14-day treatment with myricetin or myricetin plus MOR antagonist.

	Vehicle	Myricetin	Myricetin + *β*-FNA (1 *μ*g kg^−1^)	Myricetin + *β*-FNA (5 *μ*g kg^−1^)	Myricetin + *β*-FNA (10 *μ*g kg^−1^)
Plasma glucose (mg dl^−1^)	137.2 ± 5.8	112.4 ± 5.1^b^	118.4 ± 4.9^a^	125.3 ± 5.6	134.5 ± 6.2
Plasma insulin (*μ*U mL^−1^)	53.8 ± 6.2	51.6 ± 4.7	51.8 ± 5.2	52.3 ± 6.3	53.4 ± 5.9
Plasma BER (pg mL^−1^)	49.3 ± 5.2	93.5 ± 7.2^b^	92.8 ± 5.7	92.5 ± 6.1	93.0 ± 5.6
HOMA-IR score	18.2 ± 1.4	13.6 ± 1.6^a^	14.8 ± 2.1^a^	16.2 ± 1.7	17.7 ± 2.0

Fructose-fed rats received i.v. injection of myricetin (1 mg kg^−1^), thrice daily for 14 days. *β*-FNA was i.v. injected 30 min prior to administration of myricetin. The vehicle used to dissolve the tested drugs was given at the same volume. Values (mean ± SEM) were obtained from seven rats. ^a^ *P* < .05 and ^b^ *P* < .01 compared to the values of the vehicle-treated group, respectively.

**Table 2:** Quantification of the protein expression of specific insulin receptor-related signaling mediators in soleus muscles of fructose-fed rats receiving 14-day treatment with myricetin or myricetin plus MOR antagonist.

Relative units	Fructose-fed rats		
	Vehicle	Myricetin	Myricetin + *β*-FNA
IR	1.00 ± 0.07	1.04 ± 0.06	0.99 ± 0.05
IRS-1	1.01 ± 0.06	1.18 ± 0.05^a^	1.02 ± 0.08
p85	1.01 ± 0.05	1.21 ± 0.07^a^	1.08 ± 0.04
Akt	1.00 ± 0.08	1.15 ± 0.06^a^	0.96 ± 0.09
AS160	1.01 ± 0.07	1.17 ± 0.04^a^	1.03 ± 0.06

Fructose-fed rats received i.v. injection of myricetin (1 mg kg^−1^), thrice daily for 14 days. *β*-FNA (10 *μ*g kg^−1^) was i.v. injected 30 min prior to administration of myricetin. The vehicle used to dissolve the tested drugs was given at the same volume. Values (mean ± SEM) were obtained from 5 rats. ^a^ *P* < .05 represents the level of significance compared to the values of the vehicle-treated group.

## References

[1] Ong KC, Khoo H-E (1997). Biological effects of myricetin. *General Pharmacology*.

[2] Ko C-H, Shen S-C, Lee TJF, Chen Y-C (2005). Myricetin inhibits matrix metalloproteinase 2 protein expression and enzyme activity in colorectal carcinoma cells. *Molecular Cancer Therapeutics*.

[3] Ong KC, Khoo H-E (1996). Insulinomimetic effects of myricetin on lipogenesis and glucose transport in rat adipocytes but not glucose transporter translocation. *Biochemical Pharmacology*.

[4] Ong KC, Khoo H-E (2000). Effects of myricetin on glycemia and glycogen metabolism in diabetic rats. *Life Sciences*.

[5] Liu I-M, Liou S-S, Lan T-W, Hsu F-L, Cheng J-T (2005). Myricetin as the active principle of Abelmoschus moschatus to lower plasma glucose in streptozotocin-induced diabetic rats. *Planta Medica*.

[6] Liu I-M, Tzeng T-F, Liou S-S, Lan T-W (2007). Myricetin, a naturally occurring flavonol, ameliorates insulin resistance induced by a high-fructose diet in rats. *Life Sciences*.

[7] Granberry MC, Hawkins JB, Franks AM (2007). Thiazolidinediones in patients with type 2 diabetes mellitus and heart failure. *American Journal of Health-System Pharmacy*.

[8] Su CF, Chang YY, Pai HH, Liu IM, Lo CY, Cheng JT (2005). Mediation of beta-endorphin in exercise-induced improvement in insulin resistance in obese Zucker rats. *Diabetes/Metabolism Research and Reviews*.

[9] Su C-F, Chang Y-Y, Pai H-H, Liu I-M, Lo C-Y, Cheng J-T (2004). Infusion of *β*-endorphin improves insulin resistance in fructose-fed rats. *Hormone and Metabolic Research*.

[10] Tzeng T-F, Liu I-M, Cheng J-T (2005). Activation of opioid *μ*-receptors by loperamide to improve interleukin-6-induced inhibition of insulin signals in myoblast C 2C12 cells. *Diabetologia*.

[11] Tzeng T-F, Lo C-Y, Cheng J-T, Liu I-M (2007). Activation of *μ*-opioid receptors improves insulin sensitivity in obese Zucker rats. *Life Sciences*.

[12] Cheng J-T, Liu I-M, Hsu CF (2003). Rapid induction of insulin resistance in opioid *μ*-receptor knock-out mice. *Neuroscience Letters*.

[13] Liu I-M, Liou S-S, Cheng J-T (2006). Mediation of *β*-endorphin by myricetin to lower plasma glucose in streptozotocin-induced diabetic rats. *Journal of Ethnopharmacology*.

[14] Adams JU, Paronis CA, Holtzman SG (1990). Assessment of relative intrinsic activity of mu-opioid analgesics in vivo by using *β*-funaltrexamine. *Journal of Pharmacology and Experimental Therapeutics*.

[15] Matthews DR, Hosker JP, Rudenski AS, Naylor BA, Treacher DF, Turner RC (1985). Homeostasis model assessment: insulin resistance and beta-cell function from fasting plasma glucose and insulin concentrations in man. *Diabetologia*.

[16] Rodríguez E, Pulido N, Romero R, Arrieta F, Panadero A, Rovira A (2004). Phosphatidylinositol 3-Kinase activation is required for sulfonylurea stimulation of glucose transport in rat skeletal muscle. *Endocrinology*.

[17] Björnholm M, Zierath JR (2005). Insulin signal transduction in human skeletal muscle: identifying the defects in Type II diabetes. *Biochemical Society Transactions*.

[18] Muoio DM, Newgard CB (2008). Mechanisms of disease: molecular and metabolic mechanisms of insulin resistance and beta-cell failure in type 2 diabetes. *Nature Reviews Molecular Cell Biology*.

[19] Carvalho E, Rondinone C, Smith U (2000). Insulin resistance in fat cells from obese Zucker rats—evidence for an impaired activation and translocation of protein kinase B and glucose transporter 4. *Molecular and Cellular Biochemistry*.

[20] Song XM, Ryder JW, Kawano Y, Chibalin AV, Krook A, Zierath JR (1999). Muscle fiber type specificity in insulin signal transduction. *American Journal of Physiology*.

[21] Kramer HF, Witczak CA, Taylor EB, Fujii N, Hirshman MF, Goodyear LJ (2006). AS160 regulates insulin- and contraction-stimulated glucose uptake in mouse skeletal muscle. *Journal of Biological Chemistry*.

[22] Guillemin R, Vargo T, Rossier J (1977). *β*-endorphin and adrenal corticotropin are secreted concomitantly by the pituitary gland. *Science*.

[23] Lin JG, Chang SL, Cheng JT (2002). Release of beta-endorphin from adrenal gland to lower plasma glucose by the electroacupuncture at Zhongwan acupoint in rats. *Neuroscience Letters*.

[24] Liu IM, Cheng JT Mediation of endogenous *β*-endorphin in the plasma glucose-lowering action of herbal products observed in type 1-like diabetic rats.

[25] Reichard P, Nilsson BY, Rosenqvist U (1993). The effect of intensive treatment of diabetes on the development and progression of long-term complications in insulin-dependent diabetes mellitus. *The New England Journal of Medicine*.

